# Phenotypic characterization and comparative transcriptomics of evolved *Saccharomyces cerevisiae* strains with improved tolerance to lignocellulosic derived inhibitors

**DOI:** 10.1186/s13068-016-0614-y

**Published:** 2016-09-20

**Authors:** Olivia A. Thompson, Gary M. Hawkins, Steven W. Gorsich, Joy Doran-Peterson

**Affiliations:** 1 Department of Microbiology, University of Georgia, Athens, GA 30602 USA; 2 Department of Biology, Central Michigan University, Mount Pleasant, MI 48859 USA

**Keywords:** *Saccharomyces*, Fermentation, Bioethanol, Lignocellulose, Inhibitor tolerance, Transcriptomics

## Abstract

**Background:**

Lignocellulosic biomass continues to be investigated as a viable source for bioethanol production. However, the pretreatment process generates inhibitory compounds that impair the growth and fermentation performance of microorganisms such as *Saccharomyces cerevisiae*. Pinewood specifically has been shown to be challenging in obtaining industrially relevant ethanol titers. An industrial *S. cerevisiae* strain was subjected to directed evolution and adaptation in pretreated pine biomass and resultant strains, GHP1 and GHP4, exhibited improved growth and fermentative ability on pretreated pine in the presence of related inhibitory compounds. A comparative transcriptomic approach was applied to identify and characterize differences in phenotypic stability of evolved strains.

**Results:**

Evolved strains displayed different fermentative capabilities with pretreated pine that appear to be influenced by the addition or absence of 13 inhibitory compounds during pre-culturing. GHP4 performance was consistent independent of culturing conditions, while GHP1 performance was dependent on culturing with inhibitors. Comparative transcriptomics revealed 52 genes potentially associated with stress responses to multiple inhibitors simultaneously. Fluorescence microscopy revealed improved cellular integrity of both strains with mitochondria exhibiting resistance to the damaging effects of inhibitors in contrast to the parent.

**Conclusions:**

Multiple potentially novel genetic targets have been discovered for understanding stress tolerance through the characterization of our evolved strains. This study specifically examines the synergistic effects of multiple inhibitors and identified targets will guide future studies in remediating effects of inhibitors and further development of robust yeast strains for multiple industrial applications.

**Electronic supplementary material:**

The online version of this article (doi:10.1186/s13068-016-0614-y) contains supplementary material, which is available to authorized users.

## Background

Lignocellulosic biomass such as softwood is an abundant sustainable source for production of biofuels such as bioethanol. Annual softwood production varies from 8.5 to 89 million cubic meters in various countries [1–3]. Being a currently farmed forest product, there are established infrastructure and available waste streams for use. Softwoods are also advantageous in that the hemicellulose contains little xylose and arabinose alleviating the need for pentose fermenting microorganisms [4]. Work on softwood fermentation has been ongoing for a number of years with fermentations reaching high theoretical maximum ethanol yields, but only with low solids loading [5–7]. Due to its recalcitrant nature, the biomass must undergo physical and chemical treatment to release fermentable sugars. This pretreatment produces byproducts that are toxic and detrimental to growth and metabolic activity of the fermenting microbe through a variety of known and unknown mechanisms [8–10]. Inhibitors consist of phenolic compounds released from lignin as well as sugar degradation products such as furans and weak acids released from cellulose and hemicellulose [11]. Furans, such as hydroxymethylfurfural (HMF) and furfural, halt growth and ethanol production by inhibiting dehydrogenases, inducing membrane instability, disrupting mitochondria, and damaging DNA [10, 12, 13]. Weak acids, such as acetic acid, inhibit glycolytic enzymes and aromatic amino acid import [14, 15]. They also act as uncouplers disrupting the proton motive force and depleting ATP reserves [16, 17]. There is limited understanding of the effects of phenolics due to the heterogeneity of lignin, however, phenolics have been shown to induce membrane instability, act as uncouplers, and cause reactive oxygen species (ROS) damage [18–20]. Complex interactions of inhibitors also lead to synergistic effects that are not well understood [21]. These effects are further complicated by other stressors including high osmolarity of biomass solids loading, high temperature, and increasing ethanol concentrations. Therefore, further understanding of the effects of inhibitors will aid in the development of microorganisms with improved tolerance to inhibitors, which will be required for effective lignocellulosic fermentation.

*Saccharomyces cerevisiae* are widely employed for the production of several industrial products including first-generation bioethanol due to their high fermentative ability, ethanol tolerance, and rapid growth under anaerobic conditions. One hurdle that persists for the development of large scale ethanol production from lignocellulose is inhibition of yeast fermentation by furans, weak acids, and phenolics [22, 23]. Previous work has shown naturally occurring strains isolated from specific environments such as industrial settings possess a high level of inhibitor tolerance with varying degrees of fermentation performance for ethanol production, but details about improved tolerance or performance were not investigated and many strains remain uncharacterized [24–26].

To understand stress tolerance of yeast in response to inhibitors and to identify the molecular basis for improved tolerance, previous scientific investigations have employed genetic knockouts and microarray analysis to look at sensitivity to different compounds and transcriptional changes in response to exposure. The affected cellular processes include central carbon metabolism, pentose phosphate pathway, and cell membrane biosynthesis [8, 27–29]. Other important genes include transcriptional regulators, multidrug transporters, and alcohol and aldehyde reductases [27–30]. In addition to natural isolates, examinations into engineered strains have involved adaptive engineering and overexpression of genes identified from microarray analysis and genetic knockouts to improve stress tolerance. Examples include overexpression of *ADH6* and *ZWF1*, an alcohol and glucose-6-phosphate dehydrogenase, which increases HMF and furfural resistance, respectively [27, 31]. Although studies have focused on HMF, furfural, and acetic acid and investigated genetic targets and resistance to single inhibitors, few have identified genetic mechanisms for resistance to the synergistic effects of the interactions between different inhibitors found in biomass hydrolysates used for fermentation. Understanding of the effects of multiple inhibitors on yeast is critical since industrial yeast that ferment lignocellulosic hydrolysates will be exposed to multiple inhibitors at the same time. Filling in these knowledge gaps of these systems is a necessary step in understanding robustness and resistance, and can advance the development of industrially relevant novel strains for bioethanol production.

Previously, we developed evolved strains of *S. cerevisiae* through directed evolution and adaptation that were able to produce ethanol in high solids pine fermentations in the presence of unabated inhibitors [32, 33]. In this study, we identify differences in phenotypic stability of evolved strains that exhibit different fermentation capabilities influenced by culturing conditions. Comparative transcriptome analysis of both strains cultured under inhibitory conditions containing 13 compounds or none, revealed 52 genes that potentially account for the ability to perform in high solids pine fermentations, of which only six have been previously shown to be directly involved in inhibitor tolerance. To further confirm the transcriptome analysis findings, comparative RT-PCR was performed on key genes identified to compare expression levels of both evolved strains. As a result of higher mitochondrial gene expression, differences in mitochondrial morphology were also investigated.

The results of this study advance the understanding of stress tolerance of *S. cerevisiae* in response to biomass-derived inhibitory compounds. Characterization of our evolved strains has identified multiple novel genetic targets for improving mechanisms underlying yeast resistance to the synergistic effects of multiple inhibitors. Moreover, the improved growth characteristics of the evolved strains correlate with improved cellular integrity by observing a rescue of mitochondrial integrity. These data also have direct implications for further development of robust yeast strains for multiple industrial applications.

## Methods

### Growth of yeast strains

Strains GHP1 and GHP4 were obtained as previously described [33]. Each strain was grown for 24 h with 200 rpm shaking at 37 °C in yeast extract peptone dextrose (YPD) only medium containing 20 g/L peptone, 10 g/L yeast extract, and 20 g/L glucose (Sigma-Aldrich, St. Louis, MO) and separately in YPD medium supplemented with inhibitor mixture (YPDI). YPDI medium was prepared by the addition of 13 inhibitory compounds to YPD at a concentration based on 12 % dw/v pine wood hydrolysate [32, 33] (Table 1). YPD flasks at a volume of 50 mL were inoculated with 2 × 10^6^ cells from a glycerol freezer stock and YPDI flasks were inoculated with 5 × 10^7^ cells. Cellular growth rate is slower in YPDI, therefore the larger inoculum size was used for YPDI cultures to enable equivalent cell densities for pine fermentation inoculation.

**Table 1:** Concentrations (g/L) of each inhibitory compound in YPDI media

Acids		Furans		Aromatics	
Acetic acid	2.000	Furfural	1.000	3,4-DHBA	0.003
Formic acid	0.400	Furoic acid	0.020	3-HBA	0.005
Lactic acid	0.100	HMF	2.000	Benzoic acid	0.015
Succinic acid	0.030			Vanillic acid	0.050
Levulinic acid	0.400			Vanillin	0.020

*HMF* hydroxymethylfurfural, *DHBA* dihydroxybenzaldehyde, *HBA* hydroxybenzaldehyde

### Simultaneous saccharification and fermentation (SSF) of pine wood and analysis

Fermentations were performed using SO_2_-steam explosion pretreated Loblolly pine wood chips as previously described [32] with pretreatment conditions of 3 % w/v SO_2_ at 210 °C for 10 min. All pretreated pine wood samples were stored at 4 °C before use without any washing, pressing, or other method of inhibitor abatement. Moisture content of the biomass was determined using an IR-35 Moisture Analyzer (Denver Instrument, Denver, CO) and a mass equivalent to 17.5 % dw/v was weighed and placed into baffled 125 mL flasks and autoclaved for 20 min at 121 °C. Autoclaving may be considered as an additional pretreatment, and would likely be unnecessary if the material was not stored for extended periods. Autoclaving was conducted here to decrease any chances for contamination. Prior to cell inoculation, cellulase (Novozymes Inc, Franklinton, NC) at 15 filter paper units (FPU)/g dry pine and cellobiase (Novozymes Inc., Franklinton, NC) at 60 cellobiase units (CU)/g dry pine were combined and added in tryptone soy broth (TSB) medium containing 17 g/L casein digest, 3 g/L soybean meal digest, 5 g/L sodium chloride, and 2.5 g/L dipotassium phosphate (Difco, Detroit, MI) then filter sterilized via 0.2 µm filters. Additional TSB was added to a final concentration of 1× and the volume of the fermentation brought to 50 mL with sterile water. Cells from 24 h cultures were centrifuged at 5000 rpm for 10 min and inoculated into the fermentation media at an initial concentration of 2 × 10^7^ cells/mL, equivalent to approximately 2 g dw/L, an industrially relevant inoculum level. Fermentations were maintained at 37 °C, pH 5.0, with 200 rpm shaking.

Samples were taken from fermentations at the indicated time points and centrifuged at 14,000 rpm to separate out particulate matter. Supernatant was filtered via 0.2 µm filters and stored at −20 °C. Ethanol concentration was determined by gas chromatography (Shimadzu GC-08A, Columbia, MD) as previously described [34]. Samples were also examined for 41 different lignocellulosic derived inhibitory compounds using HPLC and HPLC–MS/MS methods [35, 36].

### Growth in model fermentation media

Growth analysis to assess the inhibitor tolerance of YPD and YPDI grown strains was performed using a Bioscreen C analyzer (Oy Growth Curves Ab Ltd, Helsinki, Finland). Cultures were grown in YPD or YPDI as described and then 4 × 10^5^ cells were inoculated into microtiter plates. The model fermentation medium in each well was comprised of TSB, 2 % w/v glucose, and 1.13X increased concentrations of inhibitory compounds to a final volume of 300 µL per well [32, 33] (Table 1). The initial pH of the medium was 5.0 and temperature was maintained at 37 °C without shaking. The optical density of 20 replicate wells per isolate and growth condition were read hourly at 580 nm with shaking only before measurement.

### Transcriptome sequencing and analysis

The transcriptomes of strains GHP1 and GHP4 were sequenced using Illumina miSeq paired end sequencing and standard methods (Illumina Inc, San Diego, CA). GHP1 and GHP4 were grown in either YPD or YPDI medium as described. After 24 h growth cell samples were centrifuged at 10,000 rpm and washed with sterile distilled water twice. Total RNA was prepared from each sample using Zymo YeaStar kit (Zymo Research Corp, Irvine, CA) following the manufacturer’s instructions. One library for each strain and growth condition was prepared, including parent XR122N in YPD. Briefly, mRNAs were isolated, cDNA synthesized, and the libraries finalized according to manufacturer’s instructions (Illumina Inc, San Diego, CA). All sequencing was performed at the University of Georgia’s Georgia Genomics Facility.

All libraries were combined and assembled to create a reference transcriptome using the Trinity pipeline [37]. Individual transcriptomes for each RNA library were assembled to determine the transcriptome of each inoculum. Differential expression and hierarchical clustering analysis was conducted using edgeR Bioconductor package [38, 39] to identify which sequences were highly expressed in YPD grown GHP4 and, YPDI grown GHP4 and GHP1. Selected parameters restricted analysis to transcripts that were significantly differentially expressed with a minimum fold change of 2 and *P* value of less than or equal to 0.05 with a false discovery-corrected statistical significance no greater than 0.001 (Additional file 1: Table S1). The three inocula capable of producing ethanol in high solids pine fermentations are referred to collectively as the performing samples; conversely, YPD grown GHP1 is referred to as the nonperforming sample. Sequences highly expressed in the performing samples but found to have relatively low expression in the nonperforming sample were annotated using NCBI blastx (https://www.blast.ncbi.nlm.nih.gov) and the *Saccharomyces* Genome Database (http://www.yeastgenome.org). Each sequence was compared to all yeast genome sequences available in the database to find the sequence with the greatest homology.

### RNA isolation and comparative C_T_ RT-PCR

To validate differential gene expression data from transcriptome analysis, samples in biological triplicate were analyzed by comparative C_T_ RT-PCR for nine target genes. GHP1 and GHP4 were grown in either YPD or YPDI medium and total RNA was prepared as described. Two microgram of total RNA was used to synthesize cDNA by reverse transcription using iScript cDNA Synthesis Kit (Bio-Rad, Hercules, CA) and then stored at −20 °C until use. The RT-PCR was carried out using SYBR Green (Life Technologies, Carlsbad, CA) as the reporter dye on a StepOnePlus Real-Time PCR System (Applied Biosystems, Foster City, CA). Each target gene was analyzed in triplicate for all samples in a MircroAmp Fast Optical 96 well plate (Life Technologies, Carlsbad, CA). No template controls (NTC) were also included. Reaction volumes of 20 µL contained 50 ng of cDNA, 200 nM of each primer, and 10 µL of SYBER Select Master Mix along with nuclease-free water. The primer sequences used in this study are described (Additional file 2: Table S2). PCR conditions were 95 °C for 2 min, and 40 cycles at 95 °C for 15 s and 60 °C for 1 min. Data were analyzed according the ΔΔC_T_ method as described in the Applied Biosystems Real-Time PCR Systems manual. Fold changes of target genes were determined after normalization to endogenous control UFD2 using GHP1 in YPD as the reference sample. Melt-curve analysis was used to determine specificity of amplification along with confirmation by presence of a single band for each primer pair in agarose gel electrophoresis (Data not shown).

### Fluorescence microscopy

Mitochondria were stained using MitoTracker Green FM (Molecular Probes, Eugene, OR). XR122N, GHP1, and GHP4 were grown overnight in 3 mL of liquid YPD with and without inhibitors at 37 °C and 230 rpm and subcultured. After growing for 8 h, mitochondria in samples of each strain were stained. Briefly, 1 mL of each yeast cell culture was placed in a 1.5 mL microfuge tube and spun at 12,000 rpm to form a pellet. The supernatants were discarded and the pellets resuspended in 1 mL of 10 mM HEPES buffer with 5 % w/v glucose and a pH of 7.4. To each microfuge tube, 0.1 μL of 1 mM stock solution of MitoTracker Green FM was added to bring the final concentration to 100 nM. The cells were mixed, covered, and incubated in the dark for 30 min at 30 °C. Cells were then pelleted at 12,000 rpm and the supernatant discarded. The pellet was suspended in 100 μL of 10 mM HEPES. Three microliters of cells was viewed using a fluorescent light microscope, Nikon Eclipse 80i (Nikon, Minato, Tokyo, Japan) and a FITC filter. The morphology of mitochondria was quantified in at least 100 cells.

## Results

### Pretreated pine wood fermentations and ethanol production

**Fig. 1:**
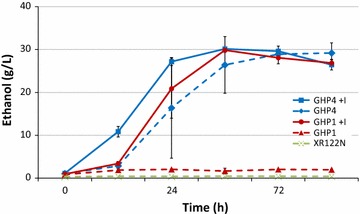
Ethanol production in 17.5 % dw/v pretreated pine (3 % w/v SO_2_ at 210 °C for 10 min) by GHP1, GHP4, and XR122N. *Solid lines* represent cultures grown with the addition of inhibitors and *dashed lines* represent cultures grown in YPD only. Data show the average of three replicate fermentations

From previous studies, evolved *S. cerevisiae* strains GHP1 and GHP4 retained improved high solids pine fermentation capabilities through isolation and culturing using inhibitor supplemented media essentially as described previously [32] (Table 1). To address the question of the stability of the fermentation phenotype, a simultaneous saccharification and fermentation process (SSF) was used with inoculation cultures of the evolved strains grown in the absence and presence of inhibitors (YPD and YPDI) and parent XR122N in YPD. When evolved strains were precultured in YPDI, GHP1 and GHP4 were consistently able to ferment the sugars present in the pine wood fermentation media to ethanol (Fig. 1). Conversely, when precultured in YPD lacking inhibitors, only GHP4 was successfully able to produce ethanol. GHP1 performed at a level similar to that of the original parent strain, XR122N (Fig. 1). GHP1 grown in YPDI and GHP4 grown in either YPD or YPDI reached maximum ethanol concentrations much higher than those obtained by GHP1 grown in YPD only and XR122N grown in YPD. GHP1 grown in YPD only reached 2 g/L of ethanol and is comparable to the performance of XR122N in YPD.

To better understand the inhibitory environment of the pine wood fermentations, 41 inhibitory compounds were measured for initial and final concentrations for any changes that occurred during fermentation. Out of the 41 compounds, only 25 were detected at quantifiable levels and the initial and final concentrations of these are presented (Table 2). Concentrations of 12 of these compounds had only minor fluctuations during the course of fermentation. Seven compounds had major decreases in concentrations: lactic acid, maleic acid, 4-hydroxybenzaldehyde, vanillin, benzoic acid, HMF, and furfural. For furfural, HMF, vanillin, and 4-hydroxybenzaldehyde, the observed decrease in concentration was greater in the successful fermentations compared to the unsuccessful fermentation. Five compounds showed considerable increases in concentration: succinic acid, 2-furoic acid, levulinic acid, 3-hydroxy-4-methoxycinnamic acid, and vanillic acid. Succinic and vanillic acids had greater increases in successful fermentations compared to the unsuccessful fermentation, with levulinic acid exhibiting the opposite trend. One compound, *o*-toluic acid, showed a decrease in successful fermentations with an increase in the unsuccessful fermentation (Table 2).

**Table 2:** Concentrations of inhibitory compounds at the start and finish of pine fermentations

Compound	Inocula^a^	Initial^b^	Final^b^	Change^b^	Percent change (%)
Decreasing compounds					
Hydroxymethylfurfural	P	1150.00	120.00	−1030.00	−89.6
	N	1370.00	930.00	−440.00	−32.1
Furfural	P	910.00	280.00	−630.00	−69.2
	N	1130.00	900.00	−230.00	−20.4
Vanillin	P	21.90	1.24	−20.67	−94.3
	N	27.47	13.82	−13.65	−49.7
Lactic acid	P	327.00	176.93	−150.07	−45.9
	N	343.33	209.33	−134.00	−39.0
Benzoic acid	P	44.03	34.83	−9.20	−20.9
	N	42.80	31.33	−11.47	−26.8
4-Hydroxybenzaldehyde	P	3.72	0.27	−3.46	−92.7
	N	4.21	2.08	−2.13	−50.6
Maleic acid	P	8.13	6.48	−1.66	−20.3
	N	8.27	1.43	−6.84	−82.7
3,4-Dihydroxybenzaldehyde	P	3.01	2.09	−0.93	−30.6
	N	3.07	3.04	−0.03	−1.0
Itaconic acid	P	1.77	1.59	−0.18	−10.2
	N	1.67	1.38	−0.29	−17.4
Increasing compounds					
Succinic acid	P	53.73	184.33	+130.60	243.1
	N	59.10	131.60	+72.50	122.7
Vanillic acid	P	30.93	57.10	+26.17	84.6
	N	27.35	42.10	+14.75	53.9
Levulinic acid	P	497.00	545.00	+48.00	9.7
	N	408.00	596.00	+188.00	46.1
2-Furoic acid	P	24.20	45.05	+20.85	86.2
	N	21.70	43.83	+22.13	102.0
3-Hydroxy-4-methoxycinnamic acid	P	8.48	14.03	+5.56	65.4
	N	4.10	7.38	+3.28	80.0
4-Hydroxybenzoic acid	P	2.79	4.35	+1.56	55.9
	N	2.37	4.33	+1.96	82.7
Glutaric acid	P	2.75	4.10	+1.35	49.1
	N	2.52	3.67	+1.15	45.6
2,5-Dihydroxybenzoic acid	P	0.14	0.48	+0.35	242.9
	N	0.07	0.18	+0.11	157.1
Syringic acid	P	3.55	3.86	+0.31	8.7
	N	3.09	3.13	+0.04	1.3
3,4-Dihydroxybenzoic acid	P	3.51	3.55	+0.04	1.1
	N	2.11	3.01	+0.89	42.7
Varying fluctuations					
*cis*-Aconitic acid	P	2.46	5.14	+2.68	108.9
	N	2.51	0.91	−1.60	−63.7
*trans*-Aconitic acid	P	2.49	3.04	+0.55	22.1
	N	1.40	1.03	−0.37	−26.4
*o*-Toluic acid	P	59.53	46.97	−12.57	−21.1
	N	50.15	57.45	+7.30	14.6
Fumaric acid	P	3.48	1.65	−1.84	−52.6
	N	2.43	2.68	+0.25	10.3
Homovanillic acid	P	4.32	4.01	−0.32	−7.2
	N	4.04	5.31	+1.27	31.4
Malonic acid	P	2.38	0.86	−1.52	−63.9
	N	2.29	3.70	+1.41	61.6

^a^P represents average of performing inocula, N nonperforming ^b^ mg/L

### Growth in model fermentation media

**Fig. 2:**
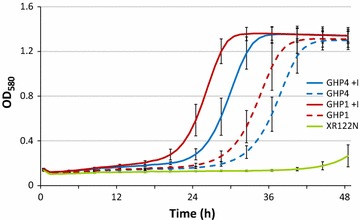
Growth of strains in model fermentation media. Concentrations of inhibitors in the media were 1.13X of those listed in Table 1. Data represent the average of 20 replicate culture wells with *error bars* showing one standard deviation from the mean

The concentration of inhibitors in the model fermentation media was increased over those in YPDI to mimic the higher concentrations of inhibitors that the cells are exposed to when inoculated into high solids pine fermentations. Both GHP1 and GHP4 cultured with and without the inhibitory compounds reached similar maximal optical densities with varying lag phases. Strains grown in inhibitor supplemented media were able to reach maximal optical density more rapidly than strains grown in YPD only (Fig. 2). GHP1 and GHP4 grown in YPD had significantly shorter lag phases than parent XR122N, indicating a much higher level of inhibitor tolerance despite being cultured without inhibitory compounds.

### Transcriptome differential expression analysis

Since GHP1 could only perform in fermentations when cultured with inhibitor supplemented media and GHP4 did not exhibit the same requirement, whole transcriptome sequencing was employed to identify differences in transcriptional response during culturing. We investigated changes in gene expression dependent on the presence/absence of the 13 inhibitory compounds that could account for differences in GHP1 and GHP4 fermentation performances. Fifty-two genes involved in various bioprocesses were identified that could account for the ability to perform in pretreated high solids pine fermentations (Table 3). Of the genes identified, nine are involved in general cellular metabolism, including alcohol and aldehyde dehydrogenases, *ADH1* and *ALD2/3*. *TDH3*, glyceraldehyde-3-phosphate dehydrogenase, had the highest increase in expression level among all sequences with a fold change of 8.2. Four genes are involved in fatty acid metabolism. At a fold change over 5, *TES1*, a peroxisomal acyl-CoA thioesterase involved in fatty acid oxidation displayed the highest fold change of the four. Three genes related to cell wall and membrane stability and function include *HES1*, an implicated regulator of ergosterol biosynthesis, and *PIR1*, which is required for cell wall stability and mitochondrial protein translocation. Eleven genes involved in transport were identified, including multidrug transporters, *PDR10* and *SGE1*. *FLC1*, a putative FAD transporter required for transport into the endoplasmic reticulum had 3.4-fold increased expression. Of known stress response genes, eight oxidative and DNA stress response genes were identified including a catalase, *CTT1*, and a glutathione transferase, *GTT1*. Seventeen of the 52 genes identified are mitochondria associated and six of them have no described function but have been previously described as part of the mitochondrial proteome: *YNL195C*, *FMP48*, *YKL187C*, *YNL208W*, *MSC1,* and *FMP16* [40, 41]. Among those with known functions were *MIP1*, the mitochondrial DNA polymerase, and *RPO41*, the mitochondrial RNA polymerase, and *MSH1*, a *MutS* homolog for mitochondrial DNA repair.

**Table 3:** Selected genes overexpressed in performing inocula

Gene	Description	FC^a^
Fatty acid metabolism		
*TES1*	Peroxisomal acyl-CoA thioesterase, involved in fatty acid oxidation	5.0
*PXA2*	Peroxisomal ATP-binding cassette transporter	2.7
*OLE1*	Fatty acid desaturase, required for proper mitochondrial functioning	2.6
*ETR1*	Thioester reductase, required for proper mitochondrial functioning	2.2
General cellular metabolism		
*TDH3*	Glyceraldehyde-3-phosphate dehydrogenase	8.2
*DFR1*	Dihydrofolate reductase, involved in tetrahydrofolate synthesis	4.3
*ALD3/ALD2*	Aldehyde dehydrogenase	4.2
*PDC6/PDC1*	Pyruvate decarboxylase	4.0
*FDH1*	Formate dehydrogenase	3.8
*TKL1/TKL2*	Transketolase	3.6
*ASN1*	Asparagine synthetase	3.1
*ARO80*	Transcriptional activator for aromatic amino acid catabolism.	2.9
*ADH1*	Alcohol dehydrogenase	2.9
Membrane/cell wall associated		
*PUN1*	Plasma membrane protein	5.2
*HES1*	Implied regulator of ergosterol synthesis	4.3
*PIR1*	Glycosylated cell wall protein, required for Apn1p mitochondrial translocation	2.3
Transport		
*SEC27*	Protein in the COPI coatomer	4.6
*ATG20*	Sorting nexin required for cytoplasm to vacuole targeting	4.4
*CHS5*	Exomer complex component	4.4
*CCC2*	Copper transporting P-type ATPase	3.5
*FLC1*	Putative FAD transporter	3.4
*PCA1*	Cadmium transporting P-type ATPase	2.9
*SUL1*	High affinity sulfate permease	2.9
*SEC22*	R-SNARE protein	2.6
*GYP7*	GTPase-activating protein	2.6
*PDR10*	ATP-binding cassette (ABC) transporter, multidrug transporter	2.6
*SGE1*	Plasma membrane multidrug transporter	1.9
Mitochondria associated		
*MRM2*	Mitochondrial *O*-ribose methyltransferase	4.6
*YNL195C*	Unknown function, part of the mitochondrial proteome	4.1
*RPO41*	Mitochondrial RNA polymerase	3.7
*FMP48*	Unknown function, part of the mitochondrial proteome	3.7
*MSH1*	MutS Homologue involved in mitochondrial DNA repair	3.4
*AEP2*	Mitochondrial protein involved in translation of OLI1 mRNA	3.4
*YKL187C*	Unknown function, part of the mitochondrial proteome	2.8
*YNL208W*	Unknown function, part of the mitochondrial proteome	2.8
*MKS1*	Pleiotropic transcriptional regulator, involved in retrograde signaling	2.7
*APJ1*	Chaperone protein involved in SUMO-mediated protein degradation	2.7
*MIP1*	Mitochondrial DNA polymerase	2.6
*MSC1*	Unknown function, part of the mitochondrial proteome	2.5
*PUT1*	Proline oxidase	2.5
*FMP16*	Unknown function, part of the mitochondrial proteome	2.5
*CYB2*	Cytochrome b2	2.3
*YNL200C*	NADHX epimerase	2.0
*DLD3*	d-Lactate dehydrogenase, part of the retrograde regulon	2.0
DNA stress response		
*IWR1*	RNA Pol II transport factor, relocates to cytoplasm upon DNA stress	4.4
*ENO1*	Enolase I, converts 2-phosphoglycerate to phosphoenolpyruvate	2.7
*TFS1*	Inhibits carboxypeptidase Y and Ira2p	2.4
*TPS2*	Phosphatase involved in synthesis of trehalose	2.0
Oxidative stress response		
*CTT1*	Cytosolic catalase T	3.7
*GTT1*	Glutathione transferase	2.7
*GAD1*	Glutamate decarboxylase	2.1
*AHP1*	Thiol-specific peroxiredoxin	2.1

^a^Fold change, average expression of performing inocula compared to nonperforming

**Fig. 3:**
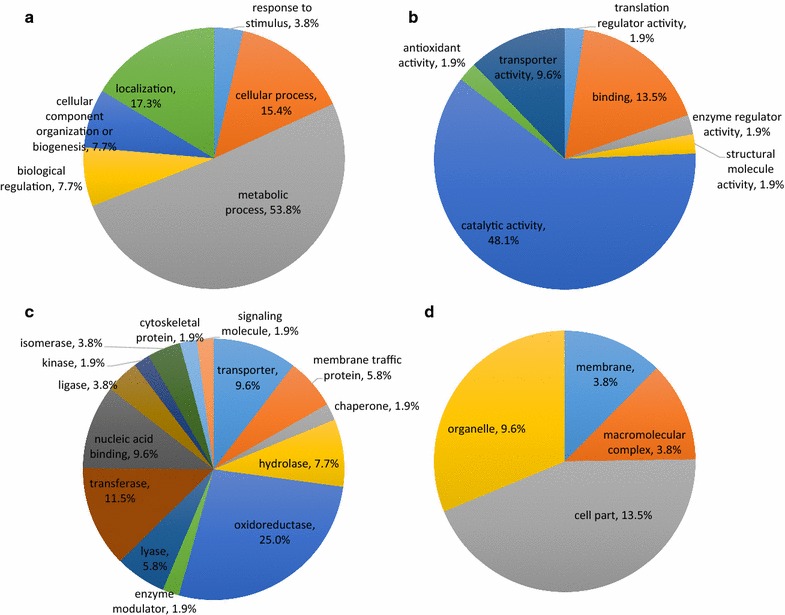
Gene ontology analysis of DEGs identified from comparative transcriptomics. Functional classification of genes is based on **a** biological process, **b** molecular function, **c** protein class, and **d** cellular component. Percentage was calculated as the number of genes involved in the corresponding process out of total number of genes identified. Performed using PANTHER v.10 [35]

Gene ontology analysis performed using PANTHER of the Gene Ontology Reference Genome Project, was used to classify the genes according to biological process, molecular function, cellular component, and protein class [42]. The 52 genes were mapped and scored to multiple annotation databases. All genes had hits to biological processes with three having hits to more than one. The majority of the hits were to metabolic processes. Other biological processes included response to stimulus, component organization, and localization (Fig. 3a). Forty-one of 52 of the genes had hits to molecular functions which included translation regulator activities, and structural molecular activities. The majority of the molecular function hits were to catalytic activities comprising 25 of the 41 genes (Fig. 3b). Protein class annotation had the largest number of categories with 48 of the 52 genes having hits. The largest category was oxidoreductases which included 13 of the genes. Other protein classes included membrane traffic proteins, hydrolases, nucleic acid binding proteins, cytoskeleton proteins, and signaling molecules (Fig. 3c). Only 16 of the 52 genes had hits to cellular components which included the categories of macromolecular complex, membrane, and organelle (Fig. 3d).

### Validation of differential expression by comparative C_T_ RT-PCR

**Fig. 4:**
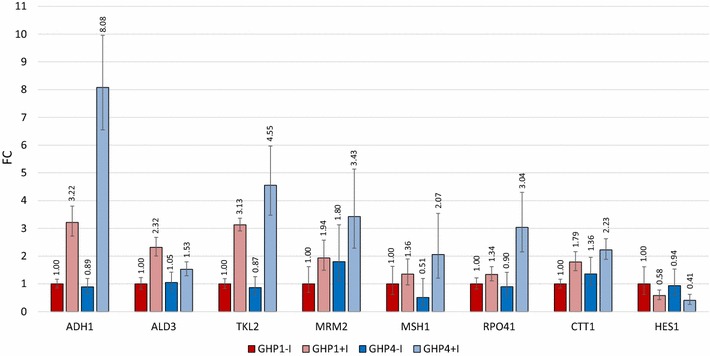
Validation of differential expression analysis by C_T_ RT-PCR. Selected genes show relative differential expression using the nonperforming sample as reference

The evolved strains GHP1 and GHP4 displayed divergent phenotypes in fermentation performances and differential expression of transcriptome profiling. We suspect that early response genes that are triggered by inhibitors during culturing before inoculation into fermentations are responsible for rapid adjustment and improved ethanol production. Thus, a subset of eight genes identified from transcriptome analysis was selected for comparative C_T_ RT-PCR. Of those, five are genes that have been previously investigated: *ADH1*, *ALD3*, *TKL2*, *CTT1,* and *HES1*. The remaining three are genes not previously shown to be related to inhibitor tolerance: *MRM2*, *MSH1*, and *RPO41*. Each target gene along with a housekeeping gene, *UFD2* as an endogenous control for normalization, was measured for both strains, GHP1 and GHP4, and in the absence/presence of inhibitors. The comparative C_T_ RT-PCR results are shown in Fig. 4. The expression profiles for the majority of the selected target genes show the same trend and consistent results from the differential expression analysis from transcriptome sequencing. For strain GHP1, all targets displayed upregulation for inhibitor grown samples compared to YPD only cultures. The same correlation was seen with GHP4 cultures in the presence and absence of inhibitors. When comparing performing samples with the nonperformer, three of the targets show increased expression: *ALD3*, *MRM2*, and *CTT1*; while the remaining five showing similar expression profiles between GHP1 and GHP4 in the absence of inhibitors: *ADH1*, *TKL2*, *HES1*, *MSH1,* and *RPO41*.

### Changes in mitochondria integrity

**Fig. 5:**
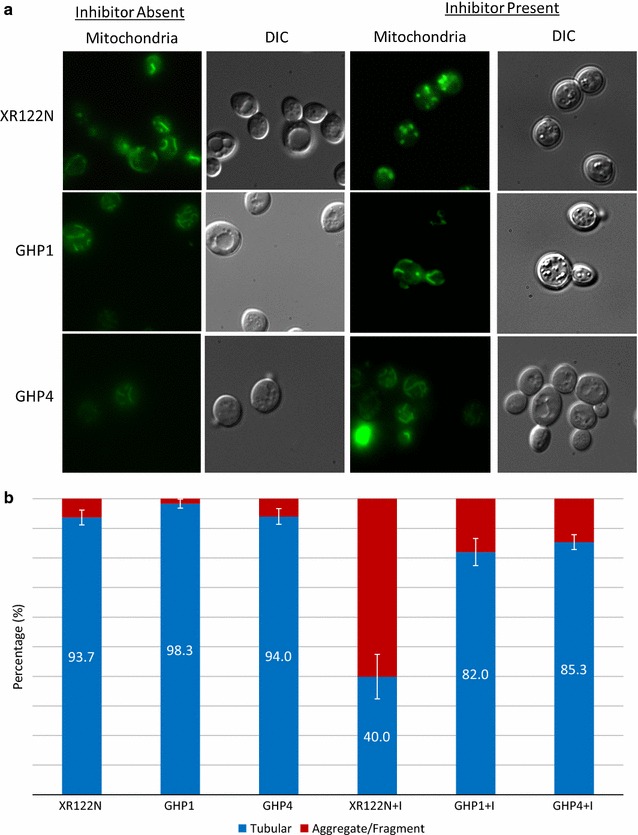
Fluorescence microscopy comparing mitochondria of performing and nonperforming samples using MitoTracker Green FM. **a** Wide field microscopy images of parent and evolved strains after 8 h growth in the presence and absence of inhibitory compounds. **b** Quantification of mitochondrial morphology. *n* = 100; experiments done in triplicate.* Blue* represents percentage of cells with intact tubular mitochondria.* Red* indicates percentage of cells with damaged/aggregated mitochondria

Of the genes identified, 32.7 % were related to mitochondria, of which nine have known functions. Fluorescence microscopy analysis was conducted to assess how mitochondrial integrity may vary between parent and evolved strains as a consequence of exposure to inhibitors. Cell cultures of parent and evolved strains were allowed to grow as described and aliquots were removed and stained with a mitochondria specific probe (Mitotracker Green FM) which allowed for visualization. Mitochondria normally appear as a tubular membrane network. For all strains grown without inhibitors, approximately 94–98 % of cells exhibited tubular well-distributed mitochondria (Fig. 5a, b). In contrast, strains grown with exposure to inhibitors displayed different mitochondria morphologies. The parent strain, XR122N, did not stain well and 60 % of visualized cells exhibited highly fragmented mitochondria. In the presence of inhibitors, both GHP1 and GHP4 strains showed mitochondria structures similar to those of cultures grown without inhibitors. Mitochondria remained fairly tubular in 82 and 85 % of the observed cells in GHP1 and GHP4, respectively (Fig. 5b).

## Discussion

Fermentation of pretreated softwood has been ongoing for decades. The most notable obstacle is the inability to have successful fermentations with increased solids beyond 10 % dw/v in which ethanol yields are drastically reduced [5–7, 43–46]. This reduction could be due in part to increased concentrations of inhibitory compounds that impair microbial growth and/or metabolic activity. A number of studies have been employed to improve fermentation performance and inhibitor tolerance. Selected strains have been cultured and co-cultured in media supplemented with low levels of hydrolysate before fermentation, which has resulted in higher ethanol yields with low solids loadings [6, 47]. Evolutionary engineering and adaptation have also been used to develop strains with increased tolerance using a defined media with one or a few inhibitors with known concentrations, or using low levels of hydrolysate of pretreated material such as wheat straw or sugar cane bagasse [48–53]. With the majority of these studies, however, very few strains developed with adaptation to pretreated biomass have been characterized or the focus is on improving xylose consumption of engineered/recombinant xylose-utilizing strains. Evolved strains GHP1 and GHP4 have been adapted to high solids loadings of pretreated pine, which has low pentose-containing hemicellulose and different inhibitor composition. Previously, we have shown that evolved strains GHP1 and GHP4 are able to reach approximately 70 % of the maximum theoretical ethanol yields in fermentations of 20 % dw/v and higher solids, without any sort of amelioration of potential inhibitory compounds [33]. While GHP4 performance was unaltered, the removal of the 13 inhibitory compounds during culturing severely impacted GHP1 performance and ethanol yields in the 17.5 % dw/v fermentations (Fig. 1). HPLC and HPLC–MS/MS analysis provided detailed insight into the inhibitor challenge strains experience during pine wood fermentation. Twenty-five different compounds were detected at quantifiable levels in the fermentation media. This does not rule out the presence of other compounds that were not assayed for presently; different pretreatment methods and the use of different biomass feedstocks would also likely lead to different suites of inhibitory compounds [11, 13, 36, 54–56]. This analysis showed that the environment is not static and that the concentration of inhibitors changes as the fermentation proceeds. Some of the changes could be due to possible differences in how the strains process toxic compounds. It has been previously shown that *S. cerevisiae* is capable of converting HMF and furfural into less toxic alcohol derivatives, which explains the decrease seen in Table 2 [30, 32, 48, 53].

In 1.13X model fermentation media, both strains in both conditions reached similar maximum optical densities with GHP1 and GHP4 grown in the absence of inhibitors having increased lag time compared to inhibitor grown cultures (Fig. 2). In comparison to previous work, the cell growth followed similar trends and reached maximum optical densities at an intermediary level than that of 1X and 1.2X model media fermentations, indicating that lag phases increase in duration as inhibitor concentrations increase [33]. GHP1’s performance implies that inhibitor tolerance alone is insufficient for fermentation of high dry weights of pine and is independent of ability to grow. In contrast the failure of XR122N performance can be attributed to severely impaired cell growth. The slight increase in growth of XR122N at 48 h could be attributed to decrease in the inhibitors by either a delay in converting HMF and furfural to less toxic derivatives, as *S. cerevisiae* has been shown to possess this trait, or evaporation of volatile compounds. It is important to note that the model fermentation may not sufficiently mimic all the stressors present in a pine wood fermentation, most notably the high solids loading which could contribute to increased osmolality stress.

The variation in ethanol production and fluctuations in inhibitor concentrations imply changes in gene regulation dependent on culturing conditions. Comparison of the transcriptomes of performing samples and the nonperforming sample identified six genes known to be involved in inhibitor tolerance from previous work in the field, along with 46 genes not previously shown to be associated with response to biomass derived inhibitors. Previous work has shown the importance of alcohol and aldehyde dehydrogenases, such as *ADH1/6* and *ALD2/3*, in the tolerance of aldehyde inhibitors, such as furans, found in biomass fermentations [28, 31, 57], which is supported by the findings in our study. The heightened expression of these genes may be responsible for the more rapid decrease in furfural, HMF, and vanillin observed in the performing sample fermentations. Transketolase (*TKL1*) is involved in the pentose phosphate pathway, and overexpression of genes in this pathway has been shown to enhance furfural tolerance *of S. cerevisiae* possibly by altering the NADH/NADPH levels inside the cell [27, 57, 58]. *FDH1*, formate dehydrogenase, may be responsible for protecting cells from formate, an inhibitory aliphatic acid known to be produced during biomass pretreatment [9]. Overexpression of formate dehydrogenase has been shown to improve fermentation performance in the presence of high concentrations of formic acid in engineered *S. cerevisiae* strains [59, 60]. Increased expression of *ARO80*, combined with *PUT1*, may alter the amino acid biosynthetic pathways in the cell and allow for more rapid ATP regeneration via the TCA cycle [28]. Overexpression of *TDH3* in performing samples may provide a more rapid NAD/NADH cofactor regeneration [58].

The overexpression of multidrug transporters has been shown to help *S. cerevisiae* survive a variety of chemical stressors, including biomass-derived inhibitors [28, 61]. The performing samples displayed heightened expression of two such genes, *SGE1* and *PDR10*. PXA2, a subunit of the peroxisomal ATP-binding cassette transporter was also more highly expressed. This protein is involved in the transport of long chain fatty acid CoA esters into the peroxisome [62]. Cellular transport has been shown in previous studies to be important for resisting the effects of HMF [28]. Integrity of cell walls and membranes are important for cells under stress from biomass-derived inhibitors as these target and disrupt these cellular structures [19]. *CHS5*, *FLC1*, *PIR1,* and *PUN1* are important for cell wall biosynthesis and functioning [63–67]. *HES1* encodes a protein that is assumed to be involved in the regulation of the biosynthesis of ergosterol, an important component of yeast cell membranes, which has been shown to be important for the tolerance of various inhibitory compounds, particularly vanillin [29, 68, 69].

When exposed to biomass derived inhibitors, cells experience both DNA damage and oxidative stress [8, 10, 13]. Eight genes associated with the response to these stressors displayed increased expression in performing samples (Table 3). Both catalase and glutathione have been shown to protect cells from reactive oxygen species [70–72]. The overexpression of glutathione transferase (*GTT1*) may increase the available glutathione pool in performing samples, while the heightened expression of cytosolic catalase (*CTT1*) may increase the cell resistance to reactive oxygen species. Contrary to our findings, *CTT1* had decreased expression upon exposure to HMF, acetic acid, or hardwood spent sulfite liquor in microarray studies using *S. cerevisiae* T2, a strain adapted for high performance in lignocellulosic biomass fermentations [73]. *AHP1*, a peroxiredoxin, has also been shown to protect cells from oxidative damage by the reduction of hydroperoxides [74]. Oxidative damage can be particularly severe to the mitochondrial DNA and causes a number of defects including loss of mitochondrial DNA and the mitochondrion itself [75].

The mitochondrion is an important organelle in eukaryotic cells that fulfills a number of roles in *S. cerevisiae* including housing a variety of metabolic activities, bioenergetics, and involvement in apoptosis [76–78]. Inhibitory chemicals found in biomass fermentations, including furfural, damage the mitochondria [13]. Seventeen sequences found in this study are connected with this organelle, suggesting an important role of mitochondria in the fermentation of high solids loadings of pine wood biomass. The increased expression of both the mitochondrial DNA and RNA polymerases suggests a role for mitochondrial replication and gene synthesis in the fermentation of high pine solids. *MSH1*, one of six *MutS* homologs in *S. cerevisiae*, is the only one that is involved in the repair and protection of mitochondrial DNA and is essential for the maintenance and functioning of the mitochondria [79]. *DLD3* and *MKS1* are part of the retrograde regulon that mediates signaling between the mitochondria and the nuclear genome and is expressed when the mitochondria are damaged [80]. In addition to their roles in fatty acid synthesis, *ETR1* and *OLE1* are important for proper formation and functioning of the mitochondria [81, 82]. *PIR1* is required for the localization of Apn1p to the mitochondria, where it functions in DNA repair and maintenance [83].

Fluorescence studies of the mitochondria have revealed distinct differences between parent strain and evolved strains. Failure of the mitochondria of XR122N to stain properly along with evidence of fragmented and aggregated structures show that mitochondria are severely impaired when exposed to inhibitors. Similar phenotypes are seen in respiratory incompetent mutants that have lost mitochondrial DNA, including *MGM1* homologue mutants that show increased sensitivity to ROS [84–86]. This damage could be a substantial reason why the parent fails to properly grow in inhibitor-supplemented media and fails to perform in high solids loading pretreated pine fermentations. In contrast, both GHP1 and GHP4 maintain fairly distributed and tubular mitochondria in both the absence and presence of inhibitors (Fig. 5). No morphological differences were observed between the performing and nonperforming samples that would support the differences seen in the transcriptomic analysis. Specifically, GHP1 cultured with and without the inhibitory compounds appear to have similar mitochondrial structures. More studies are required to understand the conditional fermentation performance of GHP1 and dependence on culturing. Nonetheless, compared to parent XR122N, both GHP1 and GHP4 show more robust mitochondria resistant to the damaging effects of biomass-derived inhibitors. Mitochondria will be further investigated to characterize differences in function. The improved robustness of mitochondria could contribute to improved processes that aid in cellular protection and repair to the damaging effects of inhibitory compounds.

## Conclusions

Evolved strains GHP1 and GHP4 have been shown to have superior high solids pine fermentation capabilities compared to parent and previously studied strains of *S. cerevisiae*. Further investigation has revealed phenotypic differences of evolved strains suggesting variable genetic content. While GHP4 exhibits constitutively inhibitor tolerant properties and successful fermentation, GHP1 shows evidence of possible on/off switches requiring the presence of inhibitors in the culturing media. Comparative transcriptomic analysis of both strains revealed a number of genes that show heightened expression in performing samples compared to GHP1 cultured without inhibitors. Some of these genes have been previously shown to be involved in inhibitor tolerance and to allow increased performance of those strains. Prominent among these are the increased expression of genes with aldehyde dehydrogenase activities which may be responsible for the more rapid removal of aldehyde inhibitors observed in fermentations with the performing samples. Additionally, the majority of the genes identified are those not previously linked to *S. cerevisiae’s* ability to withstand the negative effects of biomass-derived inhibitors. A number of genes are associated with the mitochondrion, suggesting this organelle may be vital for performance in fermentations of high concentrations of pretreated pine wood biomass. Future studies will entail further investigation of genomic changes of possible novel mechanisms adapted by evolved strains, including further mitochondria studies to understand differences in physiology and function between GHP1 and GHP4.

Recent studies have made significant advances in determining inhibitor stress response for improving yeast resistance to individual fermentation inhibitors, or a combination of a very few such as HMF, furfural, and acetic acid [27, 31, 57, 87–90]. There is still limited understanding for identifying mechanisms necessary for successful fermentation of actual pretreated biomass that contains a full suite of variable inhibitors; as it has been shown that inhibitory compounds exhibit complex interactions that lead to poorly understood synergistic effects, which creates challenges in engineering yeast. Our work addresses this problem and advances our understanding of stress resistance in yeast, specifically tolerance to biomass-derived inhibitors, and has possibly identified multiple novel targets that can improve stress resistance in *S. cerevisiae* for multiple industrial applications.

## Additional files

10.1186/s13068-016-0614-y Transcriptional analysis dataset of strains GHP1 and GHP4 in the absence and presence of inhibitory compounds. Annotation and fold change calculation from FPKM values.
10.1186/s13068-016-0614-y Primer sequences used for Comparative C_T_ RT-PCR.

## Abbreviations

RT-PCR: real time polymerase chain reaction
HPLC: high-performance liquid chromatography
CU: cellobiase units
FPU: filter paper units
dw/v: gram dry weigh per volume
HMF: hydroxymethylfurfural
TSB: tryptone soy broth
YPD: yeast–peptone–dextrose
ROS: reactive oxygen species
DEG: differentially expressed gene
FPKM: fragments per kilobase of transcript per million mapped reads
