# Supplementary material for: Phenotypic characterization and comparative transcriptomics of evolved Saccharomyces cerevisiae strains with improved tolerance to lignocellulosic derived inhibitors
Source: Biotechnol Biofuels. 2016 Sep 20;9:200. doi: 10.1186/s13068-016-0614-y (PMC5029107; doi:10.1186/s13068-016-0614-y)
Supplement: Supplementary file 2 — 10.1186/s13068-016-0614-y Primer sequences used for Comparative CT RT-PCR. [file 13068_2016_614_MOESM2_ESM.docx]

**Additional File 2: Table S2. Primer sequences used for Comparative C_T_ RT-PCR**

| Gene-Primer |  | Sequence 5’ to 3’ |
| --- | --- | --- |

| UFD2-Forward | AAT CCG GAA CTA CGG CGA TA |
| --- | --- |
| UFD2-Reverse | TTC ATG GAT CAT GGC TGT CTG T |
| TKL2-Forward | GCT GCA GGA GCC GTT AGG T |
| TKL2-Reverse | TGC AAC CCA AAT GAC TGG ATT |
| HES1-Forward | CAA CAA CGC CTG TCG CTA TG |
| HES1-Reverse | GGG AAC GAG GCC AAA ACA A |
| CTT1-Forward | GGC GTA TGT AAT ATC ACT CAA AGA ATC T |
| CTT1-Reverse | TCG CAA GTT TCG ATA GAG AAA GAG |
| MRM2-Forward | TTC CCG TCA GTA TGT GTA GTC ATT ACT |
| MRM2-Reverse | TGC CAG CCT CTG ATA TAG ATG GA |
| ADH1-Forward | CCA CGG TAA GTT GGA ATA CAA AGA TA |
| ADH1-Reverse | TCA GTG TGA CAG ACA CCA GAG TAT TTA |
| ALD3-Forward | ACG CTC TGA AGC TGG CTA ATG |
| ALD3-Reverse | CAT GTG CGC TTT CTT GAC ATC T |
| RPO41-Forward | GTG CTA TCC GCG AGT TAT TTC A |
| RPO41-Reverse | CGC ACT TTC ACC TAA CCA ATC C |
| MSH1-Forward | CAA GCA CTG AGC CCT CGA A |
| MSH1-Reverse | CAA ATC CAT CAA GTC ACG AAC ATA T |
